# Supplementary material for: larch: mapping the parsimony-optimal landscape of trees for directed exploration
Source: bioRxiv. 2025 Oct 30:2025.10.29.685337. Preprint. [Version 1] doi: 10.1101/2025.10.29.685337 (PMC12636427; doi:10.1101/2025.10.29.685337)
Supplement: Supplement 1 [file NIHPP2025.10.29.685337v1-supplement-1.pdf]

bioRxiv preprint doi: https://doi.org/10.1101/2025.10.29.685337; this version posted October 31, 2025. The copyright holder for this preprint (which was not certified by peer review) is the author/funder, who has granted bioRxiv a license to display the preprint in perpetuity. It is made available under aCC-BY 4.0 International license.

Supplementary Materials

Supplementary material, including data files, can be found in the Zenodo data repository DOI 10.5281/zenodo.17467197.

Additional figures

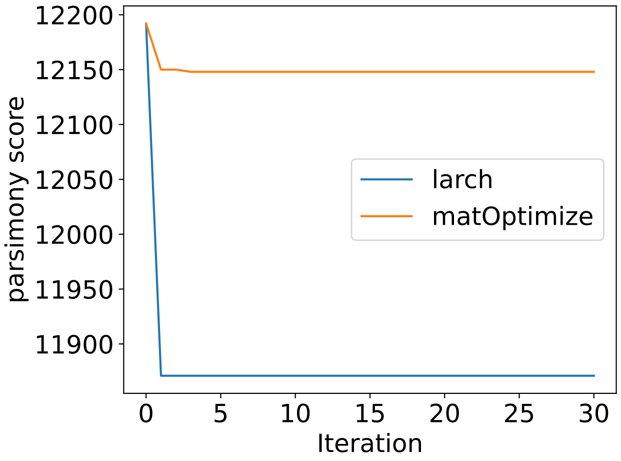

Figure S1: Comparison of the best parsimony score found by `larch` and `matOptimize` at each iteration for the Dengue dataset. The same starting history was used for each case.

bioRxiv preprint doi: https://doi.org/10.1101/2025.10.29.685337; this version posted October 31, 2025. The copyright holder for this preprint (which was not certified by peer review) is the author/funder, who has granted bioRxiv a license to display the preprint in perpetuity. It is made available under aCC-BY 4.0 International license.

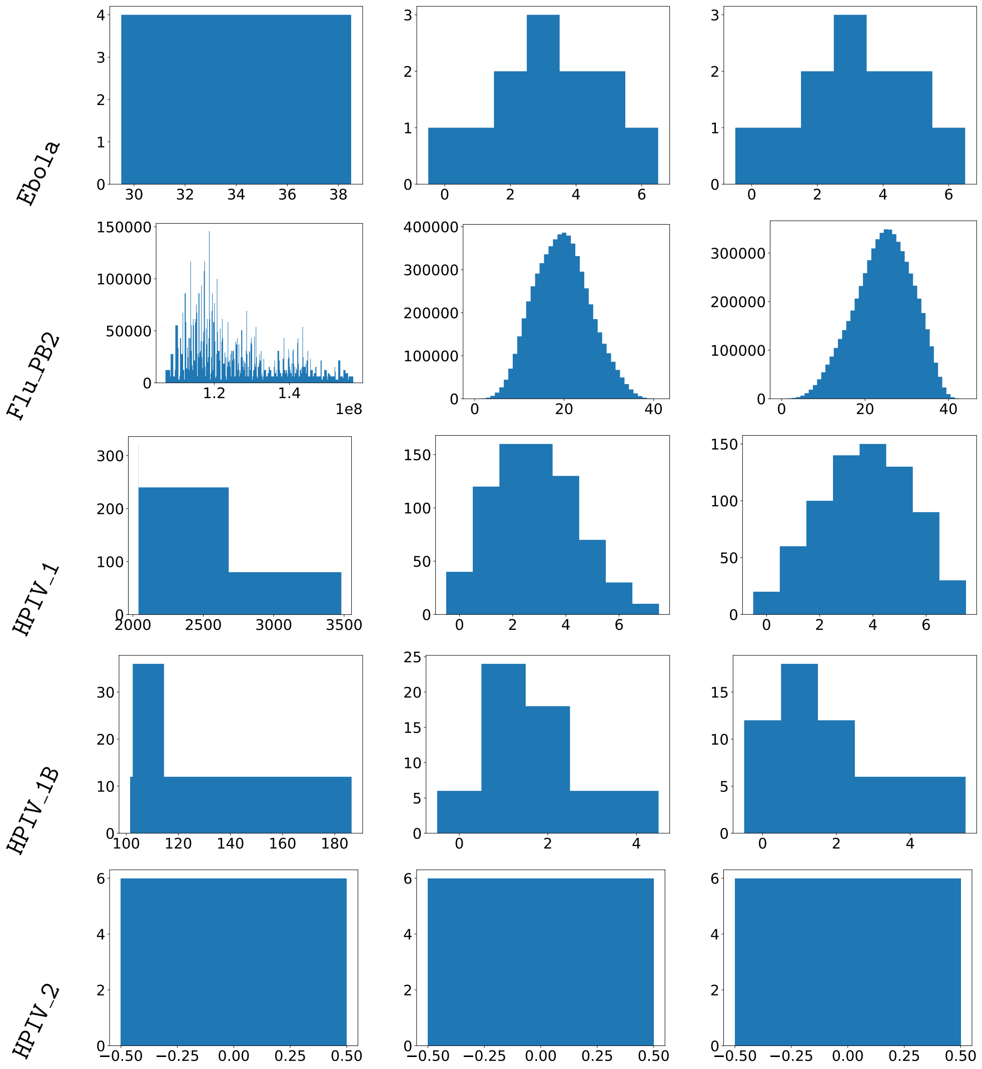

Figure S2: RF distance distributions reveal diverse clustering patterns in optimal history space across additional datasets (each row = one dataset). Left: summed RF distances for all histories. Center: pairwise RF distances to medoid history. Right: pairwise RF distances to novel history.

bioRxiv preprint doi: https://doi.org/10.1101/2025.10.29.685337; this version posted October 31, 2025. The copyright holder for this preprint (which was not certified by peer review) is the author/funder, who has granted bioRxiv a license to display the preprint in perpetuity. It is made available under aCC-BY 4.0 International license.

`larch` flags

There are a variety of optional arguments that can be used to optimize tree search using `larch`. For example, the option `move-coeff-nodes` can be used to bias the search toward SPR moves that introduce more novel features. This option specifies the factor that multiplies the number of new nodes each SPR move introduces. A factor of 0 causes `larch` to only accept SPR moves that preserve or improve parsimony. A factor of 1 or greater causes `larch` to incorporate the scaled number of new nodes into the score.

Option name	Description
`--autodetect-stoptime`	Terminate once the best parsimony score found at each iteration reaches a plateau.
`--keep-fragment-uncollapsed`	Keep edges that have no mutations associated to them, rather than collapsing them.
`--min-subtree-clade-size`	The maximum number of leaves in a subtree sampled for optimization.
`--max-subtree-clade-size`	The minimum number of leaves in a subtree sampled for optimization.
`--max-time`	Exit after fixed runtime(in minutes).
`--move-coeff-nodes`	Specify a scaling factor for how the number of new nodes contributes to SPR move score.
`--move-coeff-pscore`	Specify a scaling factor for how the change in parsimony contributes to SPR move score.
`--sample-method`	Select distribution to use for choosing a sampled history.
`--switch-subtrees`	Switch to optimizing subtrees instead of full trees after the specified number of iterations.
`--trim`	Trim the final MADAG to contain only maximally parsimonious histories.

Table S1: Optional parameters that can be used to customize the search.
